# Unmasking the potential: mechanisms of neuroinflammatory modulation by oncolytic viruses in glioblastoma

**DOI:** 10.37349/etat.2025.1002294

**Published:** 2025-02-24

**Authors:** Narimene Beder, Seyedeh Nasim Mirbahari, Mourad Belkhelfa, Hamid Mahdizadeh, Mehdi Totonchi

**Affiliations:** The First Clinical Medical College of Lanzhou University, China; ^1^Cytokines and NO-Synthases, Immunity and Pathogeny Team, Laboratory of Cellular and Molecular Biology, Faculty of Biological Science, University of Sciences and Technology “Houari Boumediene”, Algiers 16111, Algeria; ^2^Artificial intelligence and health Team, Laboratory of Cellular and Molecular Biology, Faculty of Biological Science, University of Sciences and Technology “Houari Boumediene”, Algiers 16111, Algeria; ^3^Department of Developmental Biology, School of Basic Sciences and Advanced Technologies in Biology, University of Science and Culture, Tehran 1461968151, Iran; ^4^Department of Genetics, Reproductive Biomedicine Research Center, Royan Institute for Reproductive Biomedicine, ACECR, Tehran 16635148, Iran; ^5^RoyanTuCAGene Medical and Molecular Laboratory Research Ltd., Tehran 1665894739, Iran

**Keywords:** Glioblastoma, oncolytic virotherapy, tumor microenvironment, neuroinflammation, cytokines, combined therapies

## Abstract

Glioblastoma, an aggressive and lethal brain tumor, presents enormous clinical challenges, including molecular heterogeneity, high recurrence rates, resistance to conventional therapies, and limited therapeutic penetration across the blood-brain barrier. The glioblastoma microenvironment, characterized by a dynamic interplay of cellular and non-cellular components, is a key driver of tumor growth and therapeutic resistance. Neuroinflammatory cytokines, particularly interleukins and tumor necrosis factor-alpha, play pivotal roles in this microenvironment, contributing to tumor progression and immune evasion. This review highlights oncolytic virotherapy as a promising therapeutic avenue, focusing on its potential to modulate neuroinflammatory responses, induce localized immune reactions, and deliver immunomodulatory factors directly to the tumor site. While encouraging outcomes have been observed, challenges such as overcoming the blood-brain barrier, managing host antiviral immunity, and mitigating potential risks to normal neuronal cells remain critical barriers to clinical translation. By analyzing the intricate interactions of oncolytic viruses with the glioblastoma microenvironment and synthesizing findings from preclinical and clinical trials, this review provides actionable insights into developing personalized and effective therapeutic strategies for this aggressive tumor based on oncolytic virotherapy alone or when using it combined with conventional therapies, immunotherapy, natural killer-cell therapy, chimeric antigen receptor-T cell therapy, and dendritic cell therapy.

## Introduction

Glioblastoma (GB) is the most frequent malignant and aggressive brain tumor in adults, accounting for approximately 15% of all intracranial neoplasms and 45–50% of all primary malignant brain tumors [1]. With a peak incidence in patients aged 55–85 years. The annual age-adjusted incidence rates for GB have increased in recent years to 3–6 cases per 100,000 people [2]. Its aggressive nature is reflected in its classification as a grade 4 glioma by the World Health Organization (WHO), characterized by rapid cell division, extensive infiltration into surrounding brain tissue, and the formation of abnormal blood vessels that support the tumor’s growth [3]. The intricate challenges posed by GB extend beyond its high recurrence rate and resistance to conventional therapies. The tumor exhibits molecular and genetic heterogeneity, with distinct subtypes: gliosarcoma, giant cell GB, and epithelioid GB, each presenting unique biological behaviors and therapeutic responses [4, 5]. This diversity underscores the complexity of GB and emphasizes the need for personalized treatment approaches. Despite advancements in neuro-oncology, the GB prognosis remains poor, with a median overall survival of approximately 12 to 15 months, even with aggressive treatment strategies [6]. Current treatments like surgery, radiation, and chemotherapy have limits. This emphasizes the urgent need for innovative solutions to deal with the complexities of GB’s biology. These constraints emanate from the blood-brain barrier (BBB), imposing restrictions on the effective delivery of therapeutic agents to the tumor site. This resistance, coupled with the high rate of recurrence. Oncolytic viruses (OVs) are special viruses, either natural or genetically modified, made to specifically find and kill cancer cells [7]. GB’s unique characteristics make it an ideal candidate for oncolytic virotherapy; the viruses can navigate the intricate brain environment, overcoming barriers that hamper traditional treatments [8]. GB is known for its immunologically “cold” tumor microenvironment (TME), characterized by a lack of immune cell infiltration and an immunosuppressive environment. OVs have shown potential in transforming this “cold” environment into a more “hot” and immune-responsive state. Studies have indicated that OVs can induce immunogenic cell death (ICD), leading to the release of tumor antigens and the activation of anti-tumor immune responses [9–11]. This process involves the stimulation of various immune cells, such as dendritic cells (DCs), natural killer (NK) cells, and cytotoxic T lymphocytes, which can then target and eliminate cancer cells [12]. Additionally, OVs can modulate the TME by reducing immunosuppressive factors, such as regulatory T cells (Tregs) and myeloid-derived suppressor cells, while enhancing the infiltration of effector T cells. Moreover, OVs stimulate the immune system, triggering a robust anti-tumor response that extends beyond the initial viral attack [13].

In unraveling the history of OVs in GB treatment, we witness not only a response to the shortcomings of conventional therapies but also a promising leap toward more effective and targeted interventions. The interaction between OVs and the neuroinflammatory cytokines in GB is a complex area of study. Neuroinflammation is a process involving the activation of inflammatory responses in the central nervous system (CNS), and cytokines play a crucial role in mediating these responses [14]. In the context of GB, neuroinflammation is often associated with the presence of tumor-associated macrophages (TAMs) and microglia, which release various cytokines [15]. When OVs infect GB cells, they can induce a local immune response. This response may involve the release of cytokines and chemokines, which can modulate the TME [16]. The specific effects on neuroinflammatory cytokines can vary depending on the OVs used, the characteristics of the tumor, and the host’s immune response [17]. Some OVs have been engineered to express therapeutic transgenes, including cytokines, to enhance their antitumor effects [17]. These transgenes can further influence the local cytokine TME and potentially stimulate an anti-tumor immune response. This review aims to focus centers on the intricate interplay between OVs and neuroinflammatory cytokines within the GB TME. We explore the therapeutic approaches of oncolytic virotherapy and OVs combined with other treatment protocols. We seek to contribute valuable insights that may pave the way for novel and personalized therapeutic strategies, offering renewed hope for patients grappling with this challenging malignancy.

## GB microenvironment and its impact on tumor progression

The GB TME is a critical factor in tumor progression. It promotes invasion, immune evasion, angiogenesis, and therapeutic resistance, making GB one of the most challenging cancers to treat. The GB microenvironment refers to the complex and dynamic surroundings in which GB cells, exist and interact with neighboring cells and components [18]. GB is classified as a “cold” tumor due to several features of its microenvironment that hinder an effective immune response against the tumor. GB tumors often exhibit a low rate of lymphocyte infiltration, particularly T cells, which are crucial for anti-tumor immunity [19]. This plays a role in inhibiting primary inflammation and immune responses within the CNS, where GB develops, further limiting immune cell infiltration and function [20]. Even if immune cells manage to infiltrate the tumor, the CNS has unique immunosuppressive mechanisms that can inhibit their function, contributing to the immunosuppressive nature of the GB microenvironment [21]. Additionally, GB tumors have a relatively low mutation rate and thus a low chance of neoantigen formation, which are key targets for the immune system to recognize and attack cancer cells [22]. These features collectively create an immunosuppressive TME in GB, making it less responsive to immunotherapy. The GB TME includes various cellular and non-cellular components such as tumor cells, immune cells, blood vessels, extracellular matrix (ECM), and signaling molecules. This dynamic network of interactions contributes to the unique characteristics of GB, including its invasiveness and resistance to treatment [22]. Among the cellular components, GB cells exhibit rapid proliferation, infiltration into surrounding brain tissues, and the formation of abnormal blood vessels, which are essential for sustaining their growth. Concurrently, immune cells such as TAMs, microglia, and other immune cells play a pivotal role in the microenvironment, influencing inflammation and modulating the immune response. ECM, provides structural support and regulates cell behavior [23]. Changes in the ECM composition have a profound impact on tumor invasion and progression [24]. Abnormal angiogenesis leads to the formation of leaky and disorganized blood vessels, contributing to the tumor’s nutrient supply and growth [25]. Additionally, signaling molecules such as cytokines, chemokines, and various growth factors released by tumor cells and immune cells influence immuno-inflammatory response, and the overall dynamics of the TME [26]. OVs can remodel this hostile environment by targeting tumor cells. OVs selectively infect and replicate within tumor cells, leading to their lysis. This process releases tumor-associated antigens (TAAs) into the TME, which can stimulate an immune response by exposing these antigens to immune cells, particularly DCs. OVs can also modulate immune cell dynamics and can enhance the maturation and activation of DCs, which are crucial for effective antigen presentation. By providing novel tumor antigens and promoting DC infiltration, OVs help overcome the immune tolerance typically induced by the TME. Infection with OVs can lead to the upregulation of pro-inflammatory cytokines. This change not only enhances the infiltration of cluster of differentiation 4^+^ (CD4^+^) and CD8^+^ T cells but also restores major histocompatibility complex (MHC) class I expression on tumor cells, facilitating better recognition and destruction by T cells [12, 22]. Understanding these intricate interactions is critical for developing targeted therapeutic strategies against GB.

## Neuroinflammatory cytokines: key players

**Figure 1:**
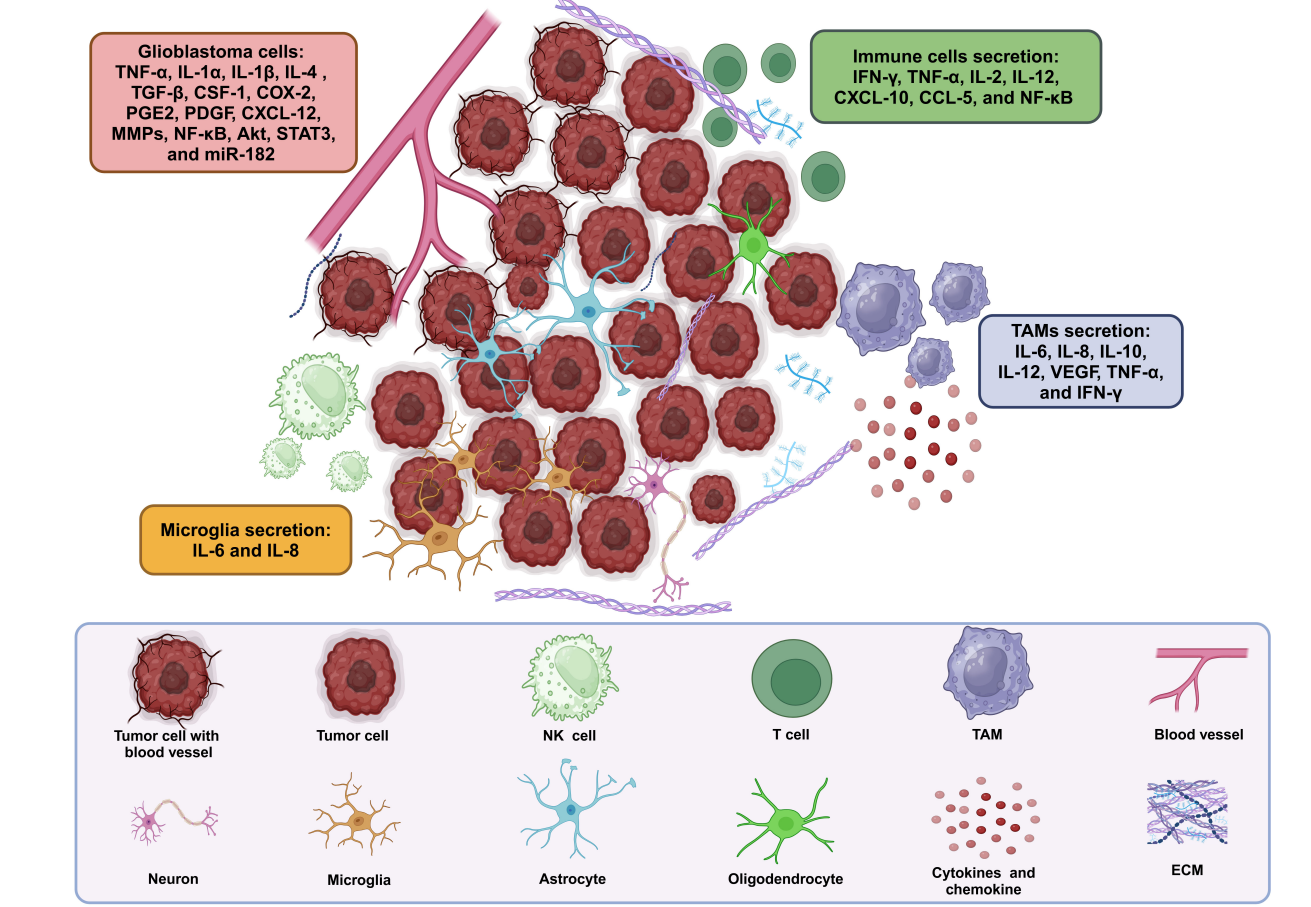
**TME: immune and other cell secretions in GB.** TAMs and microglia, along with other immune cells, secrete cytokines and chemokines such as IL-6, IL-8, IL-10, IL-12, VEGF, TNF-α, and IFN-γ, contributing to inflammation, angiogenesis, immune regulation, and tumor progression. Additionally, various other immune cells, including T cells, B cells, NK cells, and DCs, may also secrete cytokines and chemokines, further influencing immune responses and TME dynamics. Akt: protein kinase B; CCL-5: chemokine (C-C motif) ligand-5; COX-2: cyclooxygenase-2; CSF-1: colony-stimulating factor-1; CXCL-12: C-X-C motif chemokine ligand-12; DCs: dendritic cells; ECM: extracellular matrix; GB: glioblastoma; IFN-γ: interferon-gamma; IL-1α: interleukin-1α; miR-182: microRNA-182; MMPs: matrix metalloproteinases; NF-κB: nuclear factor kappa-light-chain-enhancer of activated B cells; NK: natural killer; PDGF: platelet-derived growth factor; PGE2: prostaglandin E2; STAT3: signal transducer and activator of transcription 3; TAMs: tumor-associated macrophages; TGF-β: transforming growth factor-beta; TME: tumor microenvironment; TNF-α: tumor necrosis factor-alpha; VEGF: vascular endothelial growth factor. Created in BioRender. Bahari, N. (2025) https://BioRender.com/t34f628

Neuroinflammatory cytokines play a prominent role in the intricate dynamics of the CNS during the inflammatory response in GB. Within the GB microenvironment, immune cells, particularly TAMs and microglia, are attracted and undergo activation, releasing a diverse array of cytokines. Glioma-derived cytokines, including tumor necrosis factor-alpha (TNF-α), interleukins (ILs): IL-1α and IL-1β, IL-4, IL-6, and IL-8, play pivotal roles as inflammatory mediators. These factors trigger and sustain the inflammatory cycle in GB, while also promoting carcinogenesis. They achieve this by bypassing growth suppression mechanisms, promoting angiogenesis and metastasis, resisting apoptosis, and maintaining the stemness of cancer cells [27–29]. ILs, especially IL-6 and IL-8, play a crucial role, with IL-6 being involved in the proliferation of GB cells [30, 31]. Recent studies have identified a fourth subtype of alternative M2 macrophages, activated by IL-6 and toll-like receptors (TLRs) agonists through adenosine receptors. Activation of these receptors triggers the secretion of anti-inflammatory cytokines like IL-10 and IL-12, as well as angiogenic factors such as vascular endothelial growth factor (VEGF). These findings provide additional evidence supporting the critical role of TAMs in tumor maintenance and progression [31, 32]. In vitro and in vivo studies suggest that the interaction between protein kinase B (Akt) and IL-6 depends on the autocrine/paracrine release of IL-6. This release increases Akt activity, which in turn boosts IL-6 secretion into the TME, inhibiting caspase-3. This interplay between IL-6 and Akt promotes chemoresistance and gliomagenesis [33]. IL-6 and colony-stimulating factor-1 (CSF-1) activate mammalian target of rapamycin (mTOR), promoting cell survival and the growth of alternatively activated macrophages in the TME [34]. Additionally, several studies have shown increased nuclear factor kappa-light-chain-enhancer of activated B cells (NF-κB) activity in various GB models [35]. IL-1β activates the NF-κB pathway by binding to the IL-1 receptor, leading to sustained stimulation of pro-inflammatory genes [36]. Transforming growth factor-beta (TGF-β) increases microRNA-182 (miR-182) levels, prolonging NF-κB activation in GB samples [37]. NF-κB activation in glioma cells upregulates the pro-angiogenic gene *IL-8* [38]. Inhibition of both NF-κB and signal transducer and activator of transcription 3 (STAT3) with antagonists reduces IL-6 levels, enhancing anti-invasive and cytotoxic effects in GB [39]. IL-8 is a strong pro-angiogenic factor, especially during tumorigenesis and progression [30]. It stimulates angiogenesis by promoting endothelial cell migration and the production of matrix metalloproteinases (MMPs) [40]. Targeting IL-8 in therapies could reduce tumor angiogenesis, improve permeability, hinder GB formation, and potentially enhance drug delivery. TNF-α and interferon-gamma (IFN-γ), released by immune cells, play complex roles in inflammation and tumor progression, affecting cell invasion and interactions with surrounding tissues [41]. These cytokines also impact the BBB, altering its integrity and allowing immune cell infiltration and other factors into brain tissue [42]. This disruption adds complexity to the GB microenvironment, where cytokines influence both the immune response and potentially exert anti-tumor effects, particularly IFN-γ [43]. GB cells contribute to this dynamic by showing cytokine expression heterogeneity, highlighting the complexity of the TME. Understanding how neuroinflammatory cytokines function is crucial for developing specific treatments to improve outcomes in treating GB. Figure 1 illustrates the TME in GB, highlighting the secretions of immune cells and other cellular components.

## Mechanisms of oncolytic virotherapy

### Direct oncolysis of GB cells by OVs

The therapeutic effect of oncolytic virotherapy includes direct oncolysis and induces the release of TAAs. OVs can induce cell death when they replicate, they cause direct oncolysis of GB cells, releasing new viral particles that can infect adjacent tumor cells. Some OVs are engineered to exploit specific molecular pathways in GB cells, such as mutations in protein 53 (p53) or phosphatase and tensin homolog (PTEN), which are commonly present in GB, making these cells more susceptible to viral infection and oncolysis [7, 44]. OVs have the capacity to eliminate uninfected tumor cells through a phenomenon known as the “bystander effect”. This effect may be facilitated by tunneling nanotubes that directly connect infected cells with uninfected ones, potentially leading to the apoptosis of uninfected cells by OVs. Additionally, the antigens released by OVs following the lysis of tumor cells can activate cytotoxic T lymphocytes specific to tumor antigens. Consequently, viruses and infected tumor cells can recruit innate immune cells and release cytokines such as granulocyte-macrophage (GM)-CSF, IL-12, and IL-15 [45]. These naturally released cytokines have immunomodulatory effects, promoting the maturation of antigen-presenting cells (APCs) and initiating the activation of the adaptive antitumor immune response [46, 47]. Therefore, cytokines provided by genetically modified OVs can enhance their antitumor effects by bolstering the bystander effect of OVs. Presently, a clinical trial is underway to evaluate the effectiveness of an oncolytic herpes simplex virus (oHSV) expressing IL-12 in GB [48].

### Impact of OVs on BBB

OVs can induce changes in the BBB that increase its permeability. This is crucial for allowing therapeutic agents to penetrate the brain tissue, especially in GB cases. Studies have demonstrated that certain OVs can disrupt tight junctions between endothelial cells, facilitating drug delivery to the tumor site. The ability of OVs to modify BBB permeability could improve the delivery of chemotherapeutic agents and immunotherapies directly to brain tumors, potentially leading to better treatment outcomes. The combination of OVs with other therapies can lead to necroptosis in cancer cells, which may further alter the TME and influence BBB integrity [49].

### Immune modulation of OVs in GB’s

The neuroinflammatory response in GB can be modulated by OVs by triggering an antitumor immune response and changing the TME. OVs are engineered to destroy specifically tumor cells, resulting in the targeted destruction of these cells [8, 50]. Thereby initiating an inflammatory immune response in the TME and the induction of antitumor immunity [7, 44, 48].

#### Immunoreactive cells induced by OVs

Available evidence indicates a positive correlation between tumor T cell infiltration and clinical outcomes in patients with GB, underscoring the significant therapeutic potential of T cells in GB treatment [51, 52]. Preclinical and clinical studies indicate that OVs effectively transform the “cold” immune environment of GB by triggering an inflammatory response and promoting inflammatory cell death, which leads to strong T cell activation [16, 53]. Initially, OVs replicate within tumors, causing the influx of diverse innate immune cells like NK cells and macrophages, which are linked to inflammation [16]. Following this, the pattern recognition receptors (PRRs) found on these innate immune cells can trigger a T-helper 1 (Th1) immune response by detecting either the virus or damage-associated molecular patterns (DAMPs) released when the virus lyses tumor cells. This process relies heavily on the release of IFNs, which aid in antigen presentation and the initiation of T cell-mediated adaptive antitumor immunity. As a result, augmenting T cell response against tumors through the targeting of costimulatory pathways becomes an attractive approach for GB immunotherapy [54, 55]. Incorporating T cell costimulatory molecules into OVs, such as the interaction between the costimulatory molecule OX40 and its ligand OX40L, can efficiently trigger T cell activation [56]. Delta-24-RGDOX, an oncolytic adenovirus expressing OX40L, can both recruit lymphocytes to the tumor site via ICD, like DNX-2401 (Delta-24-RGD; tasadenoturev) and activate lymphocytes that target TAAs [57, 58]. Table 1 provides information on how different OVs affect immune cells within the TME. Each OV demonstrates distinct interactions with immune cell populations, resulting in immune cell recruitment, polarization, and functional changes. These interactions play a crucial role in shaping the anti-tumor immune response and can potentially enhance the efficacy of OV therapy.

**Table 1:** The effects of various OVs on immune cells in the GB tumor microenvironment

OV	Immune cells	Effect of OV	Reference
G47D-mIL-12 (HSV-1)	TAMs	Recruiting and activating dormant monocytes into M1-like macrophages. Conv Adenovirus erting existing macrophages M2-type TAMs into the macrophages M1 phenotype.	[59]
Delta-24-RGDOX (adenovirus)	Tregs	IDO expression and Treg activation. Enhanced Ovs’ anti-tumor effects through Treg targeting.	[57, 60]
oHSV (HSV-1)	Monocytes	Recruitment and polarization of non-activated monocytes into M1-like macrophages.	[61]
Poxviral infections (wild vaccinia virus)	NK cells and DCs	Recruitment and activation of pro-inflammatory M1 macrophages and other innate immune cells like NK cells and DCs.	[62]
OV-αCD47-G4 (HSV-1)	Macrophages	Transformation of M2 macrophages into the M1 phenotype.	[63]
M010 (HSV-1)	Cytokines	Chemoattractants released by OV-infected tumor cells, such as CCL-2 and CCN1, attract TAMs and other immune cells to the tumor site.	[64]
oHSV (HSV-1)	MDSC	Increasing MDSC infiltration. Reprogramming MDSCs from a pro-tumor to an anti-tumor phenotype. The recruitment of MDSCs may depend on virus type, tumor type, and timing. High levels of MDSC infiltration are associated with resistance to OV-mediated anti-tumor effects.	[65]
NDV	CD4^+^ T cells↑ CD8^+^ T cells↑, MDSCs↓	Decreasing MDSC infiltration. Reprogramming MDSCs from a pro-tumor to an anti-tumor phenotype. The recruitment of MDSCs may depend on virus type, tumor type, and timing. High levels of MDSC infiltration are associated with resistance to OV-mediated anti-tumor effects.	[66]
Adenovirus (Delta-24-RGDOX)	IFN-γ	Induction of a M1-like phenotype in TAMs, particularly around the injection site of the OV. TAMs in this M1-like state can produce IFNs and eliminate viruses through a type I IFN-dependent mechanism. Production of IFN-γ by NK cells in response to the presence of OV.	[67]
oHSV (HSV-1)	TNF-α	Secretion of TNF-α by TAMs, has been shown to be a crucial factor in inhibiting viral replication by inducing apoptosis in OV-infected glioma cells.	[63]

CCL-2: chemokine (C-C motif) ligand-2; CCN1: cellular communication network factor 1; DCs: dendritic cells; GB: glioblastoma; HSV-1: herpes simplex virus type 1; IDO: indoleamine 2,3-dioxygenase; IFN-γ: interferon-gamma; MDSC: myeloid-derived suppressor cell; mIL-12: murine interleukin-12; NDV: Newcastle disease virus; NK: natural killer; oHSV: oncolytic herpes simplex virus; OVs: oncolytic viruses; TAMs: tumor-associated macrophages; TNF-α: tumor necrosis factor-alpha; Tregs: regulatory T cells. ↑: increase ; ↓: decrease

#### Inhibition of Tregs by OVs

Aside from generating strong antitumor T cell immunity, OVs also alter immunosuppressed T cell subsets. For instance, injecting DNX-2401 directly into tumors increases the presence of CD4^+^ and CD8^+^ T cells within the tumor while reducing the number of Tregs [68, 69]. Additional studies indicate that DNX2401 changes Tregs from being immunosuppressive to immunostimulatory by lowering the expression of the gene that encodes indoleamine 2,3-dioxygenase [70–72]. Therefore, blocking the activity of Tregs is a critical strategy to enhance antitumor T cell immunity [73].

#### Immunostimulatory cytokines produced by OVs

OVs have been shown to stimulate the immune response in GB. They can induce the production of immunostimulatory cytokines and chemokines, which in turn promote an anti-tumor immune response [74, 75]. OVs can target GB stromal cells and act on the ECM, further influencing the TME [76]. The use of OVs to stimulate the immune response in GB is an active area of research with potential implications for the development of immunotherapeutic strategies [50]. The production of immunostimulatory cytokines by OVs in the context of GB is a promising avenue for enhancing the anti-tumor immune response and warrants further investigation [77]. Cytokines represent a prevalent category of immune stimulatory factors capable of eliciting potent antitumor immunity through the recruitment and activation of T cells [78, 79]. The engineering of OVs to express cytokines such as IL-2, IL-12, and TNF has shown therapeutic efficacy in mouse tumor models [80]. Moreover, in a notable achievement, the Food and Drug Administration (FDA) approved the first oncolytic virotherapy for melanoma in 2015 [81, 82]. This modified OV includes the incorporation of the *GM-CSF* gene into a modified, non-pathogenic HSV type 1 (HSV-1) genome. The goal is to enhance the recruitment and activation of DCs, which are the most effective APCs for triggering T cell responses [76]. The oHSV carrying murine IL-12 (mIL-12) has exhibited a survival advantage in a mouse GB model [49, 83]. The findings strongly suggest that this survival benefit can be attributed to the heightened infiltration of cytotoxic T cells [84]. In a recent animal experiment, researchers used a combination treatment strategy that involved OVs expressing an IL-15 agonist in conjunction with epidermal growth factor receptor-chimeric antigen receptor (EGFR-CAR) NK cell therapy. This combined approach effectively suppressed tumor growth and significantly enhanced survival rates compared to using either treatment alone [85].

#### Shifting M2-TAM into antitumor M1-TAM by OVs

**Figure 2:**
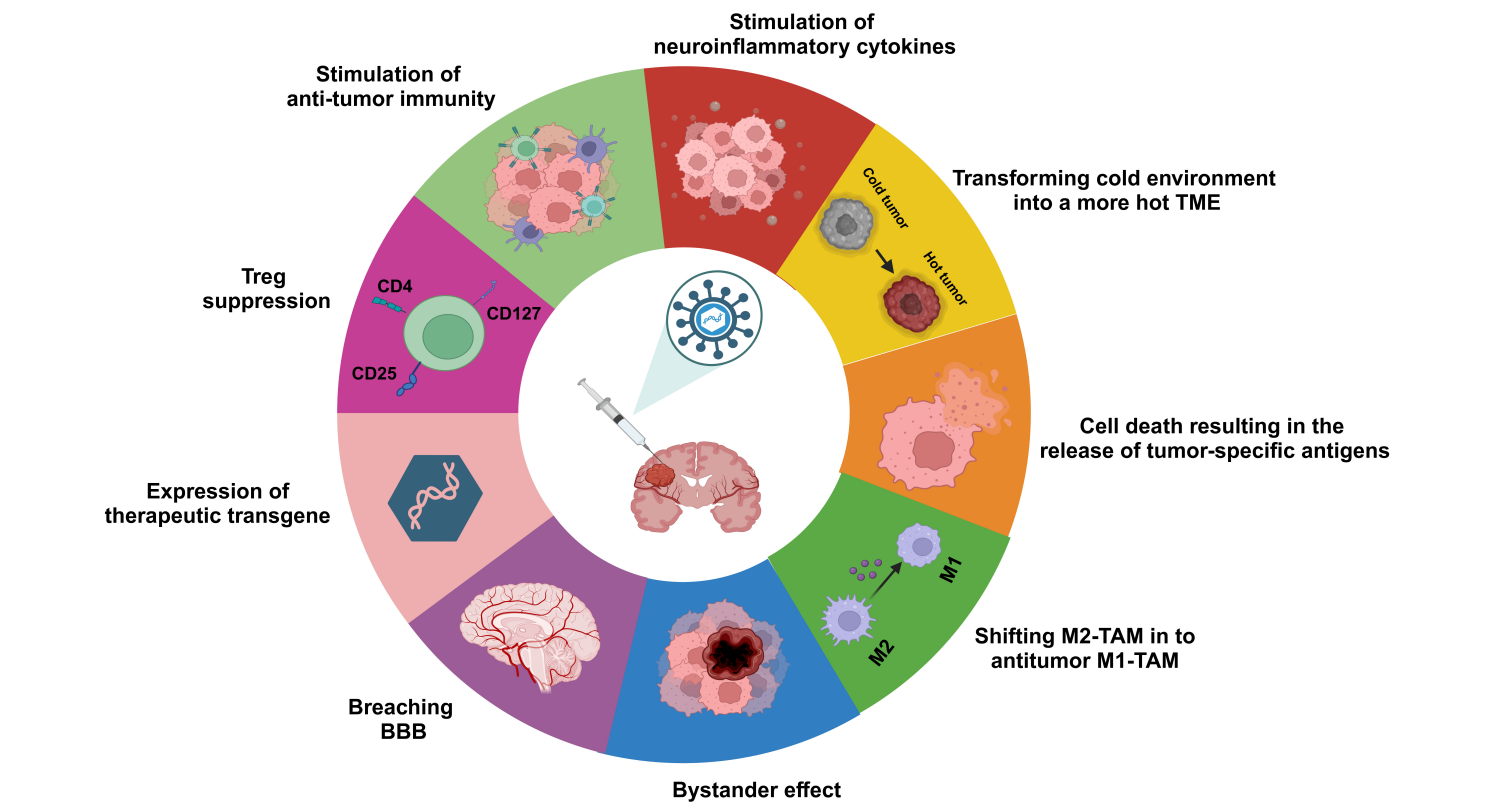
**Strategies employed by OVs in combating GB tumors.** The diagram depicts various mechanisms utilized by OVs, including enhancing anti-tumor immunity, modulating neuroinflammatory cytokines to alter the TME from a cold to a hot state, inducing cell death resulting in the release of tumor-specific antigens, shifting immunosuppressive M2-TAMs to anti-tumor M1-TAMs, leveraging bystander effects, penetrating the BBB, and suppressing Tregs. BBB: blood-brain barrier; CD4: cluster of differentiation 4; GB: glioblastoma; OVs: oncolytic viruses; TAM: tumor-associated macrophage; TME: tumor microenvironment; Tregs: regulatory T cells. Created in BioRender. Bahari, N. (2025) https://BioRender.com/r38d818

While the immune activation driven by OVs largely depends on T cells, the proper presentation of tumor antigens is also a critical factor [77, 86]. This is especially important in the GB microenvironment, where TAMs can impede lymphocyte immune surveillance. The roles of M1-TAM and M2-TAM represent two opposite ends of the spectrum in terms of the immune response [87]. M1 polarization, typically seen in the early stages of an inflammatory response, enhances T cell infiltration and initiates a subsequent adaptive antitumor response [88]. Conversely, M2-TAM in GB suppresses antigen presentation and adaptive immunity, correlating with tumor progression [89, 90]. Therefore, converting tumor-promoting M2-TAMs into tumor-killing M1-type TAMs is being explored as a potential anti-GB strategy. Studies suggest that inhibiting the expression of CD47 on tumor cell surfaces or the C-C chemokine receptor type 5 (CCR5) receptor on microglia in GB can increase the ratio of M1/M2-TAMs and improve tumor phagocytosis [91, 92]. Furthermore, IL-12, recognized for its strong ability to induce IFN-γ and drive M1-TAM polarization, demonstrates remarkable synergy in triple combination therapies when incorporated into HSV (HSV-IL12). This synergy is particularly notable when combined with anti-cytotoxic T-lymphocyte antigen 4 (CTLA-4) and anti-programmed cell death-ligand 1 (PD-L1) antibodies [49, 80]. Overall, these findings indicate that polarizing TAMs towards a pro-inflammatory phenotype can establish a TME that hampers tumor progression [93, 94]. Figure 2 depicts the tactics employed by OVs to confront GB tumors.

## Oncolytic virotherapy for GB patients at various phases of clinical trials

GB represents a disease with a pressing need for innovative therapeutic approaches. The positive results seen in preclinical studies, demonstrating improved effectiveness without added toxicity, suggest that combining new OVs with radiation and chemotherapy could be a promising new treatment approach for GB cases [95]. The existing standard of care permits the incorporation of virotherapy as a novel therapy, enabling the evaluation of cooperative effects with radiation/chemotherapy without deviating from the established treatment protocol. Where GL261 cells were injected into anesthetized C57BL/6 mice, the use of oncolytic adenovirus (DNX2401) led to an increased infiltration of CD4^+^ T cells and T-bet^+^CD8^+^ T cells [49, 52]. Likewise, injecting the Zika virus (ZIKV) directly into tumors triggers an inflammatory response that activates microglia and myeloid cells. This activation boosts antigen presentation and contributes to an antitumor effect mediated by CD8^+^ T cells [96]. Numerous OVs targeting GB have progressed through different stages of clinical trials. Notable candidates such as DNX-2401 (Delta-24-RGD; tasadenoturev), Toca 511 (vocimagene amiretrorepvec), PVS-RIPO (recombinant nonpathogenic polio-rhinovirus), and G207 have reported results from phase-I clinical trials [7, 97]. DNX2401, an oncolytic adenovirus that selectively targets tumors, has demonstrated encouraging outcomes in phase-I clinical trials. Published data indicates that the median overall survival of GB patients treated with DNX2401 reached 9.5 months. Importantly, 20% of these patients survived for more than 3 years after treatment, indicating a positive and long-lasting response in a subset of individuals. Additionally, following treatment with DNX2401, the tumor site exhibited signs of inflammation and necrosis [50]. Histopathological analysis showed the infiltration of CD8^+^ T cells and T-bet^+^ cells [98]. This finding indicates that DNX-2401 not only causes tumor regression through direct oncolysis but also triggers an antitumor immune response, which is consistent with results from preclinical studies [52, 69]. Combining DNX2401 with other treatments has advanced to phase-I/II clinical trials, including trials registered under NCT02798406, NCT02197169, and NCT01956734 [99]. Toca 511, another potential treatment, is a retrovirus that carries the cytosine deaminase gene. This virus can replicate selectively in tumor cells, producing cytosine deaminase. The cytosine deaminase enzyme encoded by Toca 511 converts the prodrug 5-fluorocytosine (5-FC) into the active chemotherapy drug 5-fluorouracil (5-FU) [100, 101]. This enzymatic process enables targeted chemotherapy within the tumor, minimizing the potential for systemic toxicity linked to 5-FU [102]. In an earlier investigation, GB patients treated with Toca 511 therapy had a median overall survival of 14.4 months. Additionally, five complete remissions were notably observed [103–105]. PVS-RIPO has demonstrated encouraging outcomes in treating recurrent GB, with a median overall survival of 24 months [106, 107]. PVS-RIPO employs a dual approach to directly eliminate cancer cells while simultaneously triggering an immune response in the host. These two modes of action are intricately linked both mechanistically and biologically, as evidenced by the immunostimulatory process of ICD. ICD is a non-canonical form of cell death induced by viruses, which promotes an immune response against the antigens present in the deceased cells. This process involves apoptosis accompanied by the release of adenosine triphosphate, the proinflammatory cytokine high-mobility group box 1, and calreticulin [108]. These released molecules immediately lead to the recruitment of DCs, antigen presentation to cytotoxic T lymphocytes, and activation of gamma-delta T cells [109]. As far as we know, 2Apro is the initial enzyme produced by PVS in tumor cells it infects [106]. Another way this virus causes cell death is through 2Apro, which is a protease that cleaves the cell’s normal protein synthesis machinery, potentially resulting in cell death [110]. Additionally, there is ongoing investigation into combining pembrolizumab, a PD-L1 inhibitor with this therapy, which has progressed to phase-II clinical trials (NCT04479241). Table 2 outlines the ongoing clinical trials using OVs for treating GB [99]. Vaccinia virus (TG6002) and adenovirus (DNX2401) show tumor-specific replication and microenvironment modulation, while reovirus and HSV variants (e.g., M032) leverage immune stimulation through GM-CSF or IL-12. PVS-RIPO demonstrates effective targeting of PVS receptor (CD155)-expressing cells with reduced neurotoxicity, and parvovirus H1 combines oncolysis with immune responses during the S-phase. However, challenges remain, including the immunosuppressive GB microenvironment, limited OV delivery across the BBB, and potential off-target effects. Further research is needed to optimize combination therapies, refine delivery methods, and identify biomarkers for patient selection, ensuring safety and efficacy in clinical applications.

**Table 2:** Clinical trials that are currently underway for OVs in GB

OV	Trial number	Phases	Anti-tumor effects
Vaccinia virus TG6002	NCT03294486	Recruiting; I/II	The removal of the *TK* gene relies on actively dividing cells to supply *TK* for replication. Incorporating GM-CSF is intended to strengthen the immune response against the tumor. Introducing the *FCU1* gene, along with the prodrug 5-FC, inhibits tumor growth.
Reovirus Reolysin	NCT00528684 NCT02444546	Completed; I Active; not recruiting; I	Tropism targets tumor cells with increased RAS expression. The inclusion of GM-CSF aims to enhance the immune response against the tumor.
Herpesvirus C134 M032 G207 rQNestin	NCT03657576 NCT02062827 NCT00028158 NCT03152318	Active; not recruiting; I Recruiting; I Completed; I/II Recruiting; I	In HSV lacking the *TK* gene, replication relies heavily on actively dividing cells to provide the necessary *TK*. HSV with a deletion in the *γ34.5* gene and a mutation in *UL39* uses enzymes from actively dividing tumor cells for replication. Additionally, HSV expressing IL-12 exhibits anti-tumor effects in vivo by combining oncolysis with the stimulation of T cell-mediated immune responses.
Parvovirus H1 H-1PV	NCT01301430	Completed; I/II	Replication takes place in actively dividing cells during the S-phase. The antitumor effect results from a combination of oncolytic effects and immune responses.
Retrovirus Toca 511	NCT01470794	Completed; I	Decrease in tumor size, and suppression of tumor growth through the delivery of therapeutic genes.
PVS-RIPO	NCT03043391 NCT04479241 NCT01491893 NCT02986178	Recruiting; I Recruiting; II	The OVs demonstrate a preference for tumor cells that express high levels of the CD155. By replacing the IRES sequence with HRV2, the virus reduces neurotoxicity and improves its ability to destroy tumors.
Measles virus MV-CEA	NCT0039029	Completed; I	The OV targets tumor cells with high CD46 receptor levels, destroying them and triggering an immune response against the tumor.
Adenovirus DNX2401 DNX2240 CRAd-S-pk7	NCT01956734 NCT03714334 NCT03072134 NCT03896568	Recruiting; II Completed; I Completed; I Recruiting; I	Adenovirus containing mutations in E1B and E1A selectively targets abnormalities in the p53 or Rb pathways of tumor cells. It redirects GB cells with low coxsackievirus and adenovirus receptor expression by modifying them with RGD or EGFR. The in vivo anti-tumor effect is mainly due to oncolysis and changes in the GB microenvironment influenced by T cells and macrophages.
HSV-1 MVR-C5252 (C5252)	NCT05095441	Not yet recruiting; I	C5252 induces a potent antitumor immune response and works synergistically with traditional cancer therapies to enhance their anticancer effects. Additionally, it exhibits anti-angiogenic properties.
HSV-1 M032	NCT02062827	Recruiting; I	This OV kills tumor cells directly through its replication process and then spreads to nearby tumor cells, continuing the destruction. It also serves as a gene therapy vector, causing tumor cells to produce and release the immunity-stimulating protein IL-12. This promotes an immune response against tumor cells, enhancing the therapy’s antitumor effect. Additionally, IL-12 has an antiangiogenic effect, further contributing to the treatment’s efficacy.

5-FC: 5-fluorocytosine; CD155: polio-rhinovirus receptor; CD46: cluster of differentiation 46; EGFR: epidermal growth factor receptor; *FCU1*: flucytosine converting enzyme 1; GB: glioblastoma; GM-CSF: granulocyte-macrophage colony-stimulating factor; HRV2: human rhinovirus 2; HSV: herpes simplex virus; HSV-1: herpes simplex virus type 1; IL-12: interleukin-12; IRES: internal ribosome entry site; MV-CEA: measles virus encoding carcinoembryonic antigen; OVs: oncolytic viruses; p53: protein 53; PVS-RIPO: recombinant nonpathogenic polio-rhinovirus; RAS: rat sarcoma; Rb: retinoblastoma protein; RGD: Arg-Gly-Asp peptide; *TK*: thymidine kinase; Toca 511: vocimagene amiretrorepvec

## Oncolytic virotherapy in GB: challenges and perspectives strategy

Recent advancements in GB treatment, such as oncolytic virotherapy and immunotherapy, offer hope for patients. These approaches target GB cells while sparing healthy tissue. However, challenges remain, despite promising results, limiting the effectiveness of oncolytic virotherapy for GB.

### Restriction of the OVs distribution by the tumor ECM

The ECM composition in brain tumors is complex and markedly distinct from that of ECM in normal healthy brain tissue [24, 111]. A growing body of research has illuminated the pivotal roles played by the ECM in the development of GB. The tumor ECM has been shown to hinder the effectiveness of cancer therapies through diverse mechanisms [112, 113]. The excessive expression of ECM components, such as hyaluronic acid and collagens especially around vessels presents a large increase, establishes barriers that envelop and shield the tumor [114, 115]. GB displays elevated levels of hyaluronic acid compared to lower-grade astrocytomas, and hyaluronic acid plays a substantial role in the aggressive invasion of GB cells [24, 116]. This constraint impedes the diffusion and penetration of drugs within the tumor, consequently reducing their efficacy, including OVs. The administration of hyaluronidase, targeting hyaluronic acid, has been shown to augment the effectiveness of various therapeutic agents in patients [117, 118]. Various collagen subtypes have been studied in relation to the invasiveness of GB. Type I collagen has been shown to increase cell migration [119] and the collagen alpha-2(I) chain is upregulated in GB compared to normal brain tissue, correlating with poor progression-free and overall survival [120]. Similarly, collagen type III affects the migration and invasion of GB cell lines, with monoclonal antibodies against type III collagen inhibiting these processes [121]. Studies have demonstrated that human GB cell lines induce the expression of collagen type IV and type VI under conditions of physical compaction. Disrupting collagen, such as with β-aminopropionitrile, inhibits GB growth in orthotropic mouse models [122]. However, an excess of ECM components can reduce nutrient and oxygen delivery to the tumor, leading to tumor hypoxia and metabolic stress [123, 124]. ECM components, especially collagens, contribute to immune trapping by impeding the migration of T cells [125, 126]. This process contributes to the exclusion of effector immune cells and the dysfunction of immune surveillance [127, 128]. OVs employed for brain tumors may encounter limitations, including insufficient virus replication or inadequate virus distribution caused by barriers within TME [83, 129, 130]. An engineered HSV, incorporating the ECM modifying MMP-9, facilitated improved viral dissemination, thereby enhancing the antitumor OV effects [24, 131, 132]. In vitro studies showed that the inclusion of MMP-9 did not alter virus infectivity but led to improved tumor regression. Furthermore, validation in primary tumors confirmed MMP-9 expression and collagen reduction. It was observed that while the enhanced virus mobilization occurred, it was offset by the increased mobility of the tumor cells. Additionally, in line with the viruses examined, conducting a comparative analysis between relaxin and MMP-9 within the same OV and model system would offer valuable insights. This comparison could help determine whether these genetic modifications yield overlapping or distinct contributions to oncolytic mechanisms [133].

Novel strategies for delivering OVs are being developed to address the challenges posed by the BBB. One such method, convection-enhanced delivery (CED) has shown promise in bypassing the BBB. CED involves using a pressure gradient within a catheter to directly distribute therapeutic agents into the interstitial spaces of the CNS, as exemplified by the application of the PVS-RIPO chimera, which has demonstrated enhanced therapeutic efficacy [106]. Recent technological advancements have introduced novel bioengineering solutions to enhance OV delivery. For instance, microbubble-assisted sonoporation has been utilized to temporarily increase BBB permeability, facilitating the systemic delivery of OVs to brain tumors [134]. Neural stem cells have emerged as effective carriers for oncolytic adenoviruses, such as NSC-CRAd-S-pk7, demonstrating safety and minimal toxicity in phase-I clinical trials for glioma patients [135]. Additionally, bioengineered lymphocytes, modified through herpesvirus saimiri (HVS)-induced immortalization, are under investigation as potential OV carriers due to their ability to evade immune responses and deliver therapeutic agents to tumors [136]. The safe and effective administration of OVs is crucial for advancing virotherapy. While intratumoral injection is commonly favored due to its ability to overcome challenges related to CNS delivery and immune system evasion, the overarching aim is to develop systemic delivery methods capable of targeting both primary tumors and distant metastases [137]. Notably, GB’s infrequent metastasis outside the CNS simplifies this objective to some extent [138].

### Clearance of OVs by host antiviral immunity

The host immune response has been demonstrated to have adverse effects in several preclinical studies involving OVs, showing reduced replication and earlier clearance of the OVs, as well as decreased anti-tumor efficacy in immunocompetent subjects compared to immunocompromised ones [139]. After the virus colonizes the tumor, the host’s antiviral immune response becomes activated and mobilized to constrain virus replication and spread. This process ultimately results in viral clearance and the elimination of the therapeutic effect [140, 141]. Thus, the optimal “time window” for most OVs to initiate anti-tumoral immunity typically falls within the first 1–2 weeks of administration, before the virus is cleared. One of the notable challenges in OV immunotherapy is to find a balance between inducing beneficial anti-tumoral immunity and dealing with concurrent anti-viral immune responses. The objective is to forestall the undesirable dominance of anti-viral effector processes, which might overshadow the establishment of acquired anti-tumoral immunity [16, 76].

A strategy to evade swift viral clearance involves temporarily suppressing early immune responses [142]. As an illustration, this was evidenced in preclinical studies using a recombinant vesicular stomatitis virus (VSV) engineered to express a broad-spectrum chemokine-binding protein (herpesvirus-1 glycoprotein G) [143]. The effectiveness of OVs was heightened by suppressing the host antiviral inflammatory response, leading to a substantial extension of survival in rats with multifocal hepatocellular carcinoma [144]. Bioengineering approaches such as cloaking OVs with polyethylene glycol or cancer cell membranes have been explored to evade early immune detection and prolong systemic circulation [145]. Another innovative method involves the use of extracellular vesicles (EVs) as carriers for OVs. EVs shield the viruses from neutralizing antibodies and immune components while increasing their tumor tropism [146]. Furthermore, genome-editing tools like Clustered Regularly Interspaced Short Palindromic Repeats-associated protein 9 are being applied to engineer OVs with reduced immunogenicity or to incorporate immunosuppressive genes that temporarily dampen host immune responses while promoting anti-tumor activity [147]. These advancements represent promising avenues for addressing the challenges of immune clearance in oncolytic virotherapy.

## OVs combined with current therapies for GB treatment

### Combining OVs with conventional therapies and immune checkpoint inhibitors

OVs represent a novel form of immunotherapy that has exhibited promising results in clinical trials, particularly when combined with existing therapeutic modalities such as conventional therapy, chemotherapy, and radiotherapy, which can be utilized to improve the efficacy of OVs [148–150]. Nevertheless, identifying the most effective combinations remains a challenging aspect of current research [52, 151–153]. OVs are replication-competent viruses that achieve their therapeutic effect through direct action, lysing tumor cells, and inducing inflammatory immune responses in and around tumors. In contrast, chemotherapy is a traditional treatment for GB. However, it can be neurotoxic and has limited efficacy in treating GB [8]. Combining OVs with chemotherapy may offer a potential solution to overcome these challenges and enhance treatment outcomes [86, 154, 155]. While challenges like the BBB and the immunosuppressive TME exist, the potential benefits of combining temozolomide (TMZ) and OVs therapy in GB make it an attractive area for further research and development. This emerging approach seeks to enhance the efficacy of current treatments [151]. TMZ stands as a first-line agent for GB therapy, yet it has only marginally prolonged overall survival by a few months [156]. OVs, such as HSV, have demonstrated potential as an effective new approach to GB immunotherapy [8]. A phase-II clinical trial of G47Δ (triple-mutated, third-generation oHSV-1), revealed a survival benefit and a favorable safety profile in 19 adult patients with residual or recurrent GB after radiation therapy and TMZ. G47Δ was administered intratumorally and repeatedly for up to six doses. The primary endpoint of 1-year survival rate after G47Δ initiation was 84.2%. Regarding secondary endpoints, the median overall survival was 20.2 months after G47Δ initiation and 28.8 months from the initial surgery. On magnetic resonance imaging (MRI), enlargement of and contrast-enhancement clearing within the target lesion repeatedly occurred after each G47Δ administration, which was characteristic of this therapy. Thus, the best overall response in 2 years was partial response in one patient and stable disease in 18 patients. Biopsies revealed increasing numbers of tumor-infiltrating CD4^+^/CD8^+^ lymphocytes and persistent low numbers of Foxp3^+^ cells. This study showed a survival benefit and good safety profile, which led to the approval of G47Δ as the first OV product in Japan [156]. Furthermore, the combination of OVs and radiotherapy presents a potential treatment strategy for GB [7, 151]. Radiotherapy, along with concurrent and adjuvant TMZ chemotherapy, is the standard of care for newly diagnosed GB. Combining OVs with these treatments may further augment efficacy and antitumor activity [156].

The lytic activity of OVs results in the release of cytokines, chemokines, and immunomodulatory molecules. This process transforms an immunologically ‘cold’ tumor, characterized by a lack of infiltrating T cells, into a ‘hot’ tumor that becomes more susceptible to immunotherapy [157–159]. Combination therapy offers an additional benefit due to the limited durability seen in monotherapy with OVs. The decrease in replication and cytolytic activity of OVs, coupled with their clearance from the body, is a significant reason for treatment ineffectiveness. This highlights the potential advantage of combination therapy in improving the immunotherapeutic effectiveness of OVs [160, 161]. A key tactic used by immunotherapy treatments is the alteration of T-lymphocyte pathways, primarily through immune checkpoint inhibition. CTLA-4, a T cell receptor that inhibits T cell activation, acts as an immune checkpoint, hindering the immune response. The initial method for immune checkpoint blockade involved using monoclonal antibodies to block CTLA-4, enhancing the immune system’s ability to target cancer cells and bolstering innate tumoricidal activity [162, 163]. PD-1/PD-L1 inhibitors are among the most effective immune checkpoint inhibitors (ICIs) currently used, representing a significant advancement in treating various cancers. However, in cancers like GB that develop an immunosuppressive TME, patients often see limited benefits from monotherapy. Combining OVs with PD-1/PD-L1 inhibitors offers a potential solution to this challenge. Mechanistically, after PD-1/PD-L1 blockade, OVs can help stimulate T cell recruitment and immune activation in the TME, reducing immunosuppression and creating a more conducive environment for an effective immune response [76]. Because the combination of immune-viral therapy is a relatively new approach, much of the evidence comes from preclinical trials. However, clinical trials are now starting to emerge for combination therapies involving OVs and ICIs. For instance, phase-II clinical trials are underway for the combination therapy of adenovirus DNX-2401 and pembrolizumab in recurrent GB. However, the results of these trials have not yet been published (NCT02798406). OVs can enhance the presence of tumor-infiltrating lymphocytes (TILs) by inducing an abscopal effect on tumor cells. This phenomenon refers to a systemic response where treating a primary tumor with immunotherapy can stimulate an immune reaction against tumors at distant sites. The ICD triggered by OVs causes the release of antigens, preparing TILs for more effective antitumor immune responses [16, 164, 165]. Hence, OV therapy plays a pivotal role in creating the essential conditions for the effectiveness of immune ICIs [140]. The combination of OV therapy with ICIs has proven to be a successful approach in preclinical studies. Various OVs, both natural and engineered, that express a diverse array of chemokines and cytokines, have demonstrated encouraging outcomes [166–168]. The development of ICIs has revolutionized current cancer therapy. OVs can induce an increase in immune cells and immune checkpoint molecules within TME. In a study conducted by Chen et al. [74], vaccinia OV armed with IL-2 successfully alters the balance between cancer and immune responses, effectively modulating the cancer-immune set point. This viral construct demonstrates efficient control of multiple murine tumor models without causing adverse effects. When combined with PD-1/PD-L1 blockade, it achieves significant tumor regression in the majority of mice with significant tumor charges. This combined therapeutic approach presents a promising solution for cancers that have previously exhibited resistance to standard immunotherapy methods. In addition to immune checkpoint molecules expressed in T cells and tumor cells, OVs have also been shown to upregulate immune checkpoint molecules primarily located in NK cells [169]. Wang et al. [170], reported that oHSV-2 upregulates NK group 2A (NKG2A) expression on NK cells and human leukocyte antigen E (HLA-E) on tumor cells. They found that anti-NKG2A/anti-HLA-E antibodies enhance the antitumor effects of UV light-inactivated oHSV-2-stimulated NK92 cells both in vitro and in vivo [170]. Nakao et al. [171], reported that intra-tumoral expression of IL-7 and IL-12 using OVs increases systemic sensitivity to immune checkpoint blockade. The upregulation of immune checkpoint molecule expression can offer targets for subsequent combination therapy with ICIs in clinical studies. Large-scale phase-III trials have solidified the role of OVs, showcasing their ability not only to lyse cells for cancer-killing effects but also to induce favorable alterations in TME. To further enrich the future perspectives, it is important to highlight the potential of combination therapies tailored specifically to the challenges posed by GB. Integrating OVs with emerging strategies such as CAR-T cell therapy, bispecific antibodies, or targeted molecular inhibitors may address GB’s unique features, including its immunosuppressive TME and intratumoral heterogeneity. Early results suggest these therapies could work well together and are generally safe Table 3 [172].

**Table 3:** Key RNA OVs of interest in combination with immunotherapies in early clinical development

OV	VSV-IFN-β-NIS	VSV-GP128	VSV-GP	MG1	CVA21	Pelareorep (formerly Reolysin^®^)
Virus type	VSV	VSV	VSV	Maraba virus	Picornavirus (coxsackievirus)	Reovirus
Combination immuno-therapies	Avelumab Pembrolizumab Ruxolitinib Cemiplimab	Anti-PD-1	Ezabenlimab	Pembrolizumab Atezolizumab	Pembrolizumab Ipilimumab	Avelumab Bevacizumab Bortezomib Carfilzomib Nivolumab Pembrolizumab

Avelumab: an anti-PD-L1 immune checkpoint inhibitor; pembrolizumab: an anti-PD-1 immune checkpoint inhibitor; ruxolitinib: a Janus kinase 1/2 inhibitor; cemiplimab: an anti-PD-1 immune checkpoint inhibitor; ezabenlimab: an anti-PD-1 immune checkpoint inhibitor; atezolizumab: an anti-PD-L1 immune checkpoint inhibitor; ipilimumab: an anti-cytotoxic T-lymphocyte antigen 4 immune checkpoint inhibitor; bevacizumab: a vascular endothelial growth factor inhibitor (anti-angiogenic therapy); bortezomib: a proteasome inhibitor used in cancer treatment; carfilzomib: a second-generation proteasome inhibitor; nivolumab: an anti-PD-1 immune checkpoint inhibitor. CVA21: coxsackievirus A21; GP: glycoprotein; IFN-β: interferon-beta; NIS: sodium iodide symporter; OVs: oncolytic viruses; PD-1: programmed cell death-1; PD-L1: programmed cell death-ligand 1; VSV: vesicular stomatitis virus

### Cell therapy based treatments combined with OVs in GB treatment

#### Improving NK cell therapy through OVs

Combining OV and NK cell therapy in GB represents an emerging approach for treating this aggressive form of malignant brain tumor. OVs show promise as treatments for GB, as they have the ability to target and destroy glioma cells [7]. Indeed, NK cells are a type of immune cell with the unique ability to recognize and attack cancer cells without the need for prior sensitization. Their innate ability to target and eliminate abnormal cells, including cancer cells, makes them valuable in the context of cancer therapy [8]. The efficacy of OV-bortezomib (proteasome inhibitor) combination therapy can be enhanced by controlling the quantity of NK cells [173]. Reducing the count of endogenous NK cells or elevating the quantity of exogenous NK cells enhances the effectiveness of the antitumor treatment, the use of checkpoint blockade to overcome the immunosuppressive effect within the TME could increase the endogenous NK cell antitumor ability and the function of adoptive transferred NK cells. Therefore, it is reasonable to combine ICIs with adoptive transfer of allogeneic NK cells or CAR-transduced NK cells [174]. A brief postponement in NK cell adjuvant therapy might enable a broader spread of the virus before the initiation of adjuvant therapy, consequently enhancing antitumor efficacy [175]. Nevertheless, a substantial delay in adjuvant therapy diminishes the advantage of NK cell-induced tumor cell killing, resulting in compromised antitumor efficacy [175]. It is crucial to highlight that endogenous NK cell depletion commenced 2 days before viral injection, and the exogenous NK cell therapy was administered a few days later (3 days) following OV treatment. This timing was chosen to capitalize on the NK cell-induced alterations in TME after OV therapy. Depleting endogenous NK cells or injecting exogenous NK cells both resulted in increased survival time, although it remains unclear which of the two strategies is superior [45]. The choice between depleting endogenous NK cells and injecting exogenous NK cells should also take into account the potential negative side effects, including a weakened immune system with NK cell depletion and the risk of toxicity and inflammation associated with the injection of exogenous NK cells [175].

#### Enhancing CAR-T cell therapy with OVs

Cellular immunotherapy, including T cell therapy, has shown limited success as well [176]. This is particularly evident in the treatment of solid tumors, where T cells face challenges in infiltration and survival. Combining OVs with T cell therapy has demonstrated potential to amplify the benefits of both treatments. Recent in vivo data, for instance, indicated that an IL-7-loaded oncolytic adenovirus, when combined with B7H3-targeted CAR-T cells therapy, exhibited superior anti-tumor efficacy for GB compared to monotherapy with either treatment. The authors propose that OVs enhanced the therapeutic efficacy of CAR-T cell therapy by supplying activation signals that facilitated the infiltration of T cells into the tumor [147]. OVs and CAR-T cells can be genetically engineered to enhance their combined immunotherapeutic effectiveness. Specifically, OVs can be tailored to express the same antigen found in tumor cells that are used to construct CAR-T cells, enabling targeted killing. This strategy has been employed in the combination of CD19-CAR-T cells and OV19t, which is an OV engineered to express a truncated form of CD19. When OV19t was administered after CD19-CAR-T cells, it resulted in an enhanced immune response, with increased infiltration of both innate and adoptively transferred T cells into the tumor. Furthermore, the cytotoxic effects of CAR-T cells led to the release of the OV, which in turn promoted the expression of CD19, creating a positive feedback loop that provided a continuous target for CAR-T cell activity. OVs that are genetically modified to produce chemokines like C-X-C motif chemokine ligand-11 can help recruit T cells into the tumor. This approach, when used alongside CAR-T cell therapy, can overcome the challenges posed by the TME [177]. OVs are modified to target immune checkpoints, and when used alongside CAR-T cells, they have shown increased effectiveness in treating solid tumors [178]. Although combining OVs with CAR-T cells can enhance their antitumor effects, this approach is still in its early stages. Further improvements are necessary to ensure efficient delivery and maintain a lasting therapeutic response [158, 179].

#### Employing OVs alongside DC therapy

The combination of OVs with DCs therapy has been explored as a potential cancer treatment. OVs can induce cancer cell lysis and immune activation, triggering the release of danger signals through immunogenic cancer cell death. This process may reverse tumor immunosuppression, facilitating the expansion, activation, and recruitment of DCs and T cells in TME. The combination of OVs with DCs therapy in the treatment of GB is an area of active research. DC vaccination has been evaluated in the context of GB therapy, with studies showing variable efficacy ranging from little to significant response [180]. Furthermore, a phase-III trial has evaluated the addition of an autologous tumor lysate-pulsed DC vaccine (DCVax^®^-L) to standard therapy for newly diagnosed GB, indicating that the addition of DCVax^®^-L to standard therapy is feasible, safe, and may extend survival [181]. These findings have evaluated only DC therapy for GB. Concluding the promising results of combination therapy involving DCs and OV from this study requires further investigation.

## Conclusions

The GB TME presents a challenging landscape for effective treatment due to its immunosuppressive nature and resistance to therapies. Neuroinflammatory cytokines play a crucial role in shaping this environment, promoting tumor progression and immune evasion. Understanding these interactions is key to developing targeted therapies. OVs offer a promising approach to modulating the GB TME. They can induce tumor cell lysis, release TAAs, and trigger an inflammatory immune response, enhancing antitumor immunity. Clinical trials with OVs, including those expressing cytokines like IL-12, show potential in treating GB. Future research should focus on optimizing OV therapy for GB. This includes addressing challenges such as restricted distribution within the tumor and clearance by host immunity. Combining OVs with other therapies, such as ICIs, DCs, NK, and CAR-T cell therapy, holds promise in enhancing therapeutic efficacy. Additionally, further understanding of the interactions between OVs and the GB microenvironment is needed to develop more effective immunotherapeutic strategies. Overall, OVs represent a promising avenue for improving outcomes in GB treatment, and continued research in this area is essential for advancing cancer therapy.

## Abbreviations

5-FU: 5-fluorouracil
Akt: protein kinase B
APCs: antigen-presenting cells
BBB: blood-brain barrier
CAR: chimeric antigen receptor
CD4^+^: cluster of differentiation 4^+^
CED: convection-enhanced delivery
CNS: central nervous system
CSF-1: colony-stimulating factor-1
CTLA-4: cytotoxic T-lymphocyte antigen 4
DCs: dendritic cells
ECM: extracellular matrix
EVs: extracellular vesicles
G47Δ: triple-mutated, third-generation oncolytic herpes simplex virus type 1
GB: glioblastoma
GM: granulocyte-macrophage
HLA-E: human leukocyte antigen E
HSV: herpes simplex virus
HSV-1: herpes simplex virus type 1
ICD: immunogenic cell death
ICIs: immune checkpoint inhibitors
IFN-γ: interferon-gamma
IL: interleukin
MMPs: matrix metalloproteinases
NF-κB: nuclear factor kappa-light-chain-enhancer of activated B cells
NK: natural killer
NKG2A: natural killer group 2A
oHSV: oncolytic herpes simplex virus
OVs: oncolytic viruses
PD-1: programmed cell death-1
PD-L1: programmed cell death-ligand 1
PVS: polio-rhinovirus
PVS-RIPO: recombinant nonpathogenic polio-rhinovirus
TAAs: tumor-associated antigens
TAMs: tumor-associated macrophages
TILs: tumor-infiltrating lymphocytes
TME: tumor microenvironment
TMZ: temozolomide
TNF-α: tumor necrosis factor-alpha
Toca 511: vocimagene amiretrorepvec
Tregs: regulatory T cells

## Declarations

### Copyright

© The Author(s) 2025.
